# Supplementary material for: Biophysical basis of filamentous phage tactoid-mediated antibiotic tolerance in P. aeruginosa
Source: Nat Commun. 2023 Dec 19;14:8429. doi: 10.1038/s41467-023-44160-8 (PMC10730611; doi:10.1038/s41467-023-44160-8)
Supplement: Supplementary file 1 — Supplementary Information [file 41467_2023_44160_MOESM1_ESM.pdf]

Supplementary Information

Containing Supplementary Figures 1-10 and Supplementary Tables 1-2

"Biophysical basis of filamentous phage tactoid-mediated antibiotic tolerance in *P. aeruginosa*"

Jan Böhning[1], Miles Graham[1,2], Suzanne C. Letham[1,2], Luke K. Davis[3,4], Ulrike Schulze[5], Phillip J. Stansfeld[6], Robin A. Corey[7,8], Philip Pearce[3,4], Abul K. Tarafder[1,*], Tanmay A. M. Bharat[1,*]

[1] Structural Studies Division, MRC Laboratory of Molecular Biology, Francis Crick Avenue, Cambridge CB2 0QH, United Kingdom
[2] Sir William Dunn School of Pathology, University of Oxford, Oxford OX1 3RE, United Kingdom
[3] Department of Mathematics, University College London, London WC1H 0AY, United Kingdom
[4] Institute for the Physics of Living Systems, University College London, London WC1E 6BT, United Kingdom
[5] Cell Biology Division, MRC Laboratory of Molecular Biology, Francis Crick Avenue, Cambridge CB2 0QH, United Kingdom
[6] School of Life Sciences & Department of Chemistry, University of Warwick, Coventry, United Kingdom
[7] Department of Biochemistry, University of Oxford, Oxford OX1 3QU, United Kingdom
[8] School of Physiology, Pharmacology and Neuroscience, University of Bristol, BS8 1TD

Supplementary Figures

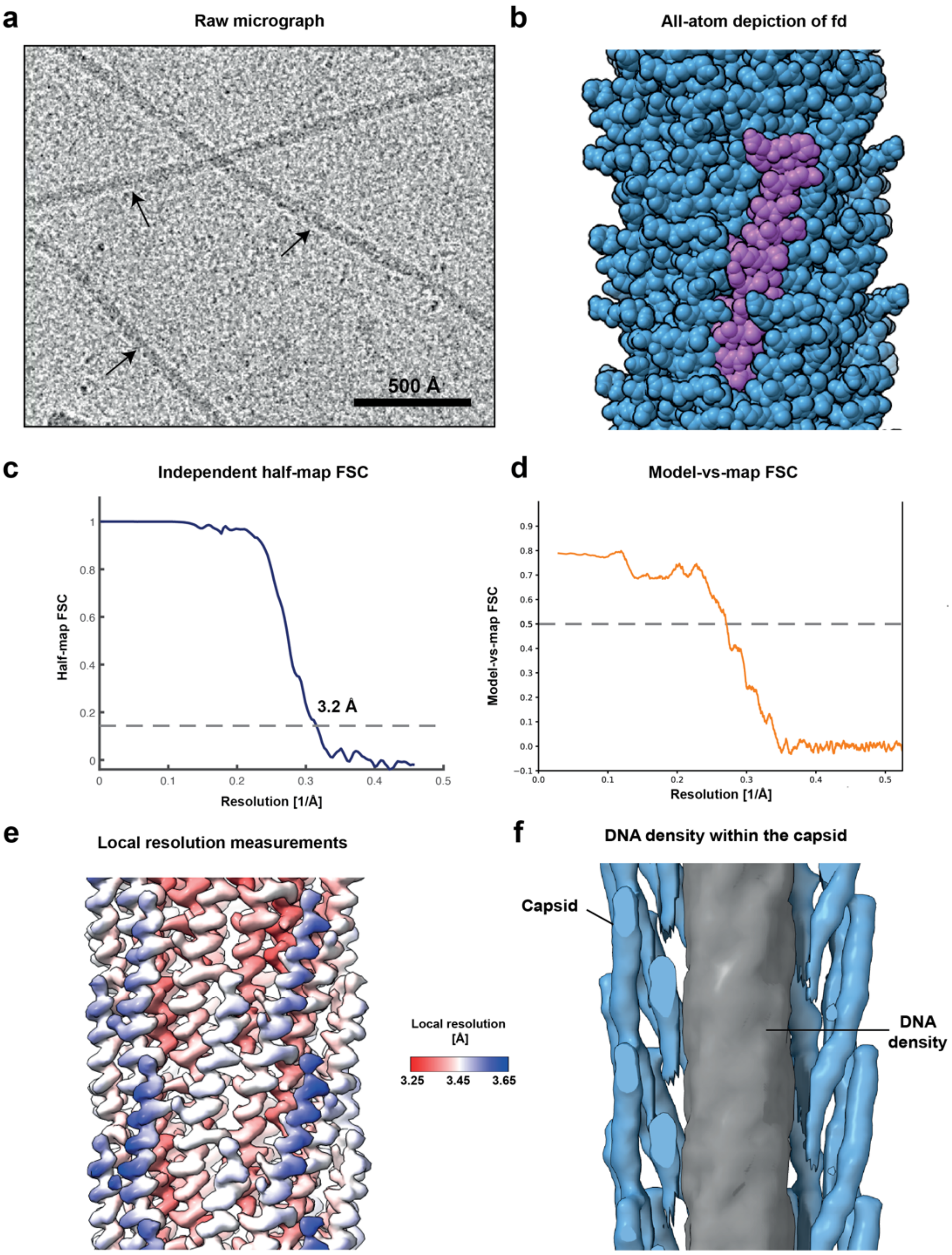

Supplementary Figure 1: Cryo-EM structure of the bacteriophage fd at 3.2 Å resolution.
a) Cryo-EM image of native fd phage specimen used for structure determination. Individual phage filaments are marked by an arrow. **b)** All-atom sphere depiction of the bacteriophage as shown in Figure 1a, with one capsid subunit marked in purple. **c)** Fourier Shell Correlation (FSC) as calculated by RELION. The 0.143 criterion used for resolution estimation is shown as a dotted line. **d**) Model vs map FSC as calculated by PHENIX. **e)** Local resolution measurement of the cryo-EM structure. Very small local resolution differences were observed in the map, indicating that most of the capsid protein is rigidly positioned in the phage. **f)** Sliced side view of cryo-EM density refined with C1 symmetry (at lower resolution). Density (2σ contour level) corresponding to capsid (blue) and DNA (grey) are highlighted. Source data for graphs are provided as a Source Data file.

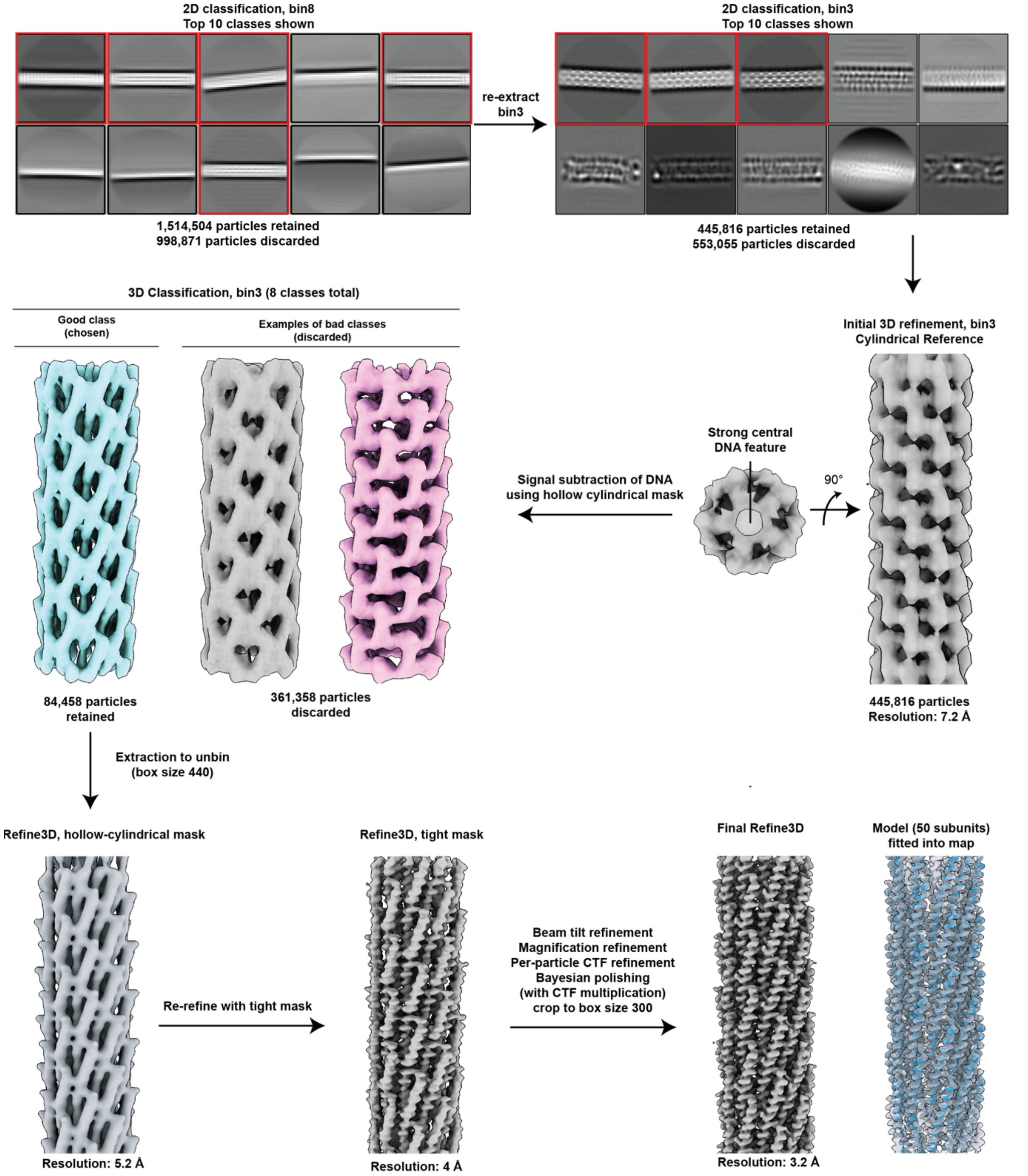

Supplementary Figure 2: Cryo-EM processing workflow for the fd bacteriophage capsid. Shown are the major 2D and 3D classes as well as 3D refinements obtained using RELION. Chosen 2D classes are respectively framed in red. Helical and C5 symmetry was applied for all 3D refinements. Maps with estimated resolutions (0.143 independent half-map FSC) of higher than 5 Å are shown B-factor-sharpened. The fit of the final capsid model, with 50 chains modelled, is furthermore shown.

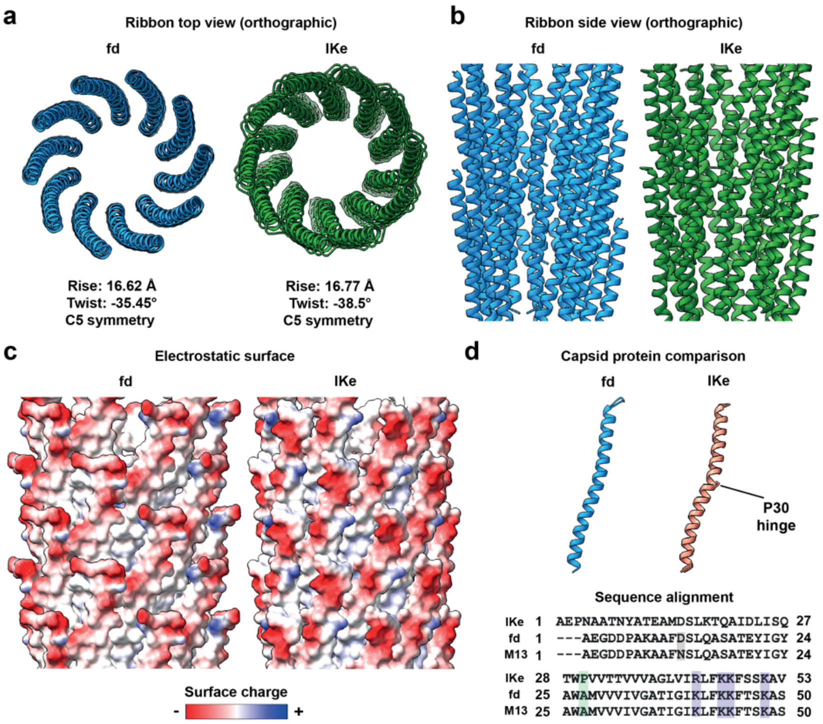

Supplementary Figure 3: Comparison of fd with the other class I bacteriophage IKe.
a) Orthographic top view of fd versus IKe, showing a higher twist in the case of IKe. **b)** Side view of fd and IKe capsids, excluding their N-terminal residues that are not resolved in the cryo-EM density. **c)** Comparison of electrostatic surface of full-length fd and IKe reveals a negatively charged capsid surface in both cases, with three negatively charged residues in the disordered N-terminus in the case of fd. **d)** While showing a similar overall morphology, a proline residue (P30) induces a hinge in the pVIII protein of IKe. A Clustal Omega sequence alignment of the major capsid proteins of IKe, fd and M13 are shown. IKe P30 is highlighted in green and positively charged residues at the C-terminus highlighted in purple. The residue differing between fd and M13 is marked in grey.

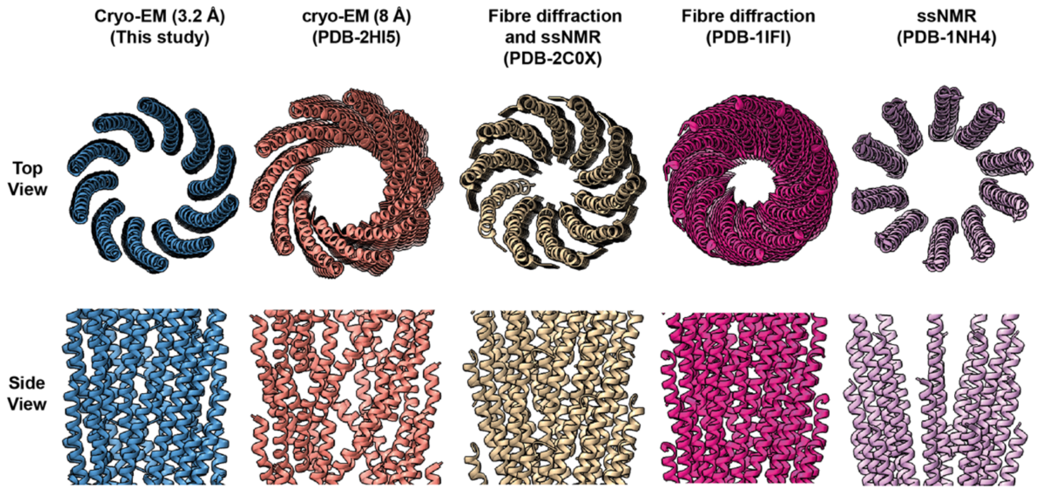

Supplementary Figure 4: Comparison of the 3.2 Å-resolution cryo-EM structure obtained in this study with previous structural models of fd.
Shown are ribbon diagrams (top and side view) of the 3.2 Å-resolution cryo-EM structure reported in this study compared to previously proposed fd capsid models based on an 8 Å-resolution cryo-EM map (PDB 2HI5), ssNMR and fibre diffraction combined (PDB 2C0X), fibre diffraction alone (PDB 1IFI), and ssNMR alone (PDB 1NH4).

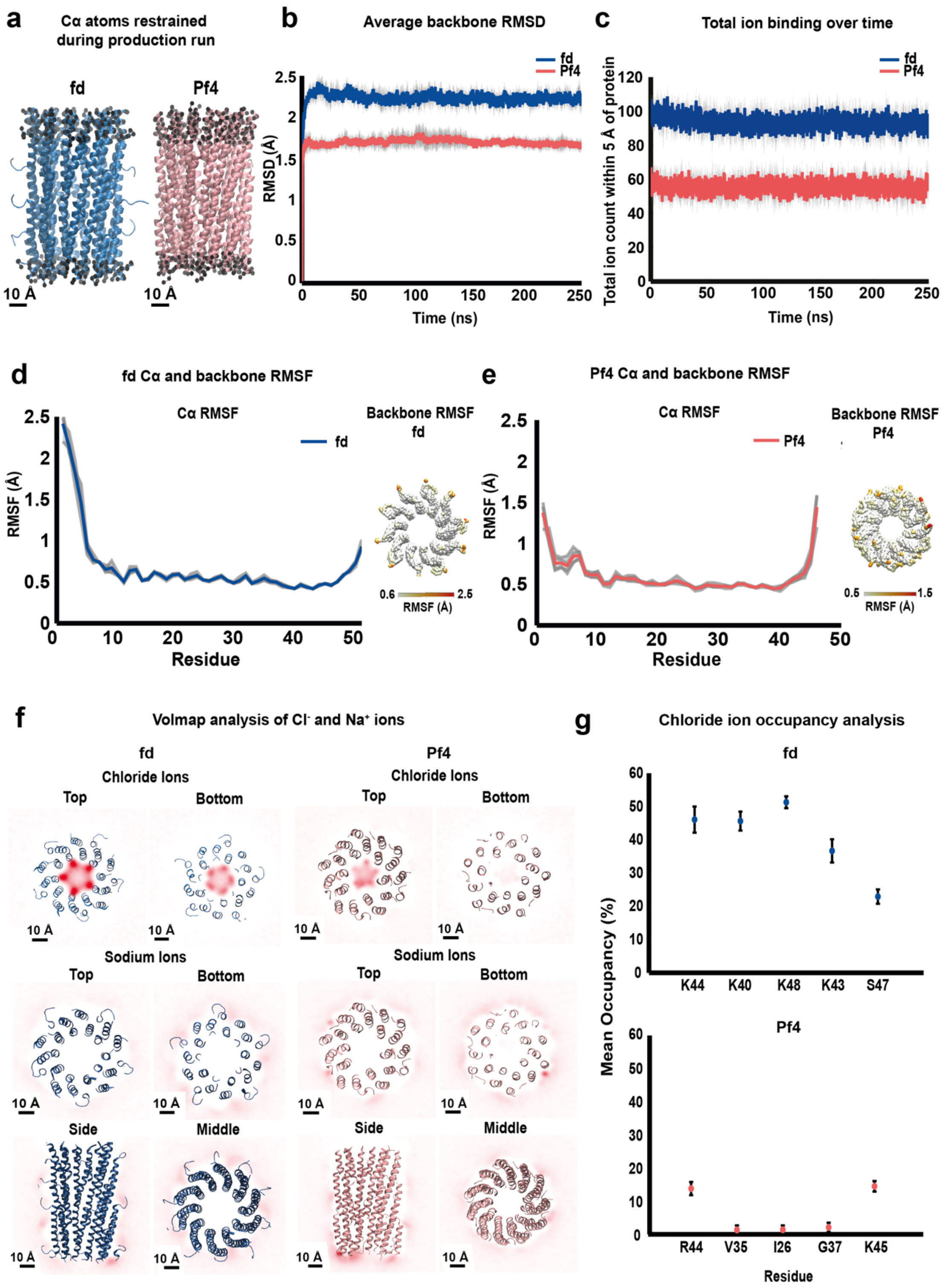

Supplementary Figure 5: Atomistic molecular dynamics simulations of fd and Pf4 capsid proteins.

a) Side view of fd (left) and Pf4 (right) showing Cα atoms restrained during simulation production runs. Restrained Cα atoms are shown as black spheres. **b)** Backbone RMSD plot of simulated systems for 250 ns in 0.15 M NaCl. Average RMSD (Å) is plotted, for fd (blue) and Pf4 (salmon) with the standard deviation plotted in grey colour. Both simulation systems are stable. **c)** Total ion binding over the course of the 250 ns simulations. Average total ions within 5 Å of fd (blue) and Pf4 (salmon) with the standard deviation plotted in grey colour. Both systems converge with respect to total protein-ion interactions. **d)** Fd Cα RMSF plot (left) and backbone RMSF values plotted on the simulated structure (right) over 250 ns of simulation in 0.15 M NaCl. Average Cα RMSF (Å) values are plotted in solid blue (left), with

individual repeats plotted in grey colour. RMSF show higher values at the N-terminus. **e)** Corresponding plots for Pf4, with average Cα RMSF values plotted in salmon (left), with individual repeats plotted in grey colour. RMSF values plotted on the simulated structure (right). **f)** Weighted ionic density of sodium and chloride ions (calculated using volmap analysis in the VMD software) averaged over all trajectory frames of fd (left) and Pf4 (right). Density of ions are shown in red. Dark red indicates higher ionic density. Volmap slice offsets used were 0.5 for side and middle, 0.28 for bottom and 0.67 for top views. There is a lack of sodium ions in the capsid lumen although they are present around the capsid on the outside. **g)** Quantification of chloride ion residue occupancy for positively charged C-terminal residues of fd (top) and Pf4 (bottom). Quantification was performed using PyLipID for ion analysis. Mean occupancy data is plotted, with error bars depicting SEM. The highest chloride ion occupancy residues are plotted for both systems, with overall higher occupancy detected for fd. Source data for graphs are provided as a Source Data file.

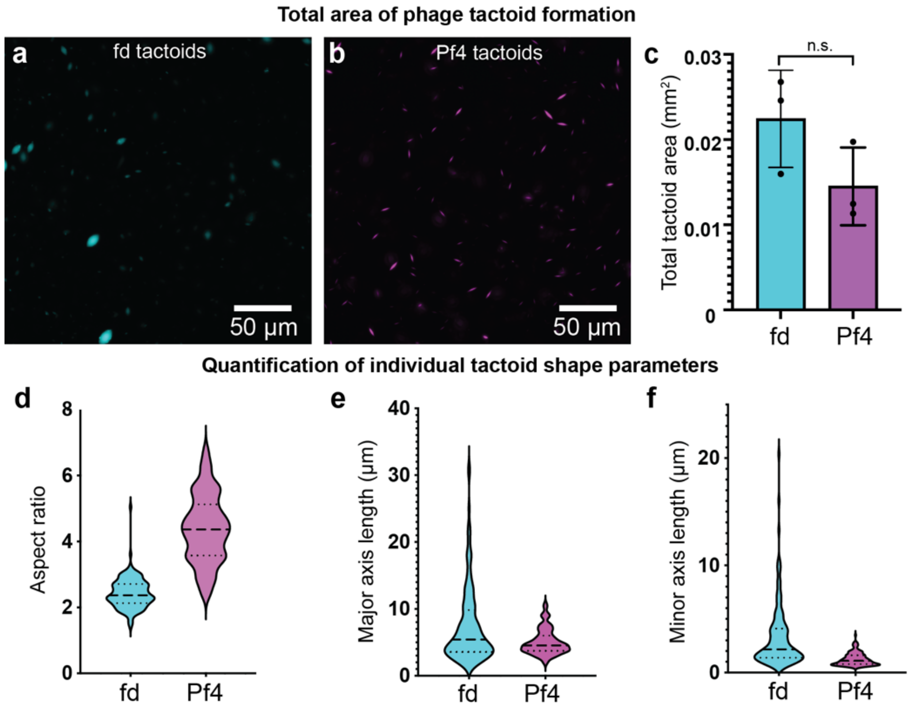

Supplementary Figure 6: Quantification of phage tactoid morphology.
a-b) Representative light microscopy images with comparable total tactoid area formed by A488-labelled fd (cyan) and Pf4 phages (magenta). **c)** Bar chart showing total tactoid area as assessed by light microscopy followed by segmentation of tactoids. Values shown are the mean of three independent experiments and error bars represent standard deviation. No significant difference was observed between fd and Pf4 droplets, P_{value}=0.1334, All p-values were calculated using an unpaired t-test. **d)** Violin plot of aspect ratios of individual tactoids. **e)** Violin plot of major axis lengths of individual tactoids. **f)** Violin plot of minor axis lengths of individual tactoids. Dotted lines indicate mean and $25^{th}/75^{th}$ percentiles. Source data for graphs are provided as a Source Data file.

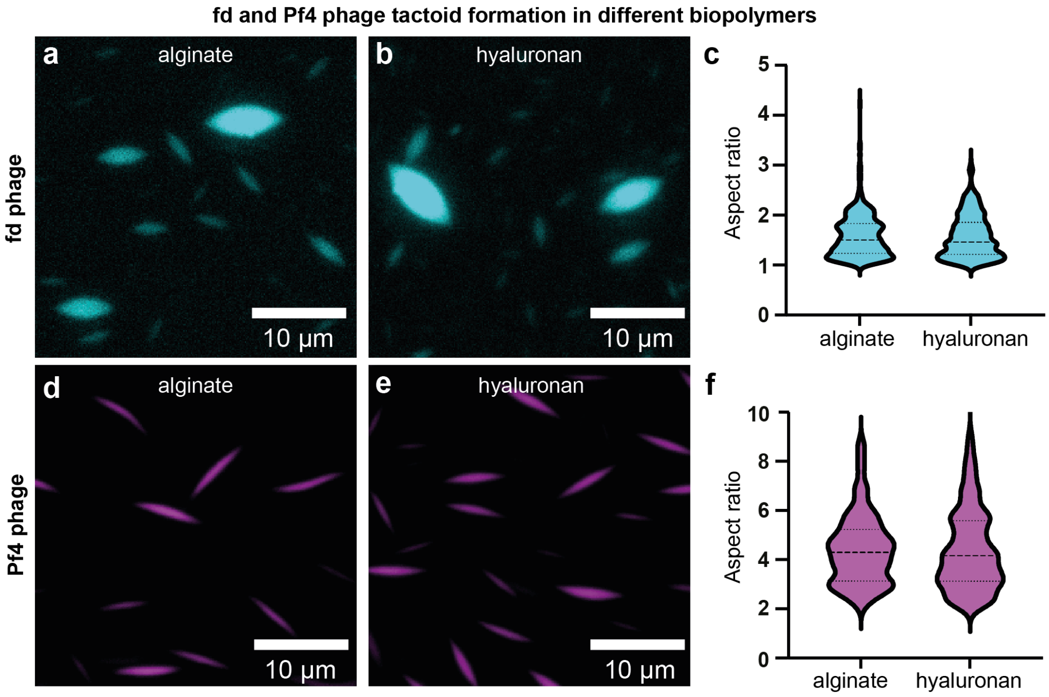

Supplementary Figure 7: Tactoid formation of Pf4 and fd in the presence of alginate versus hyaluronan.

a-b, d-e) Light microscopy images of fluorescently-labelled fd (cyan) or Pf4 (magenta) tactoids formed in the presence of either alginate or hyaluronan as crowding biopolymer. **c,f)** Violin plot of measured aspect ratios of tactoids formed in alginate versus hyaluronan. Dotted lines indicate mean and 25th/75th percentiles. Source data for graphs are provided as a Source Data file.

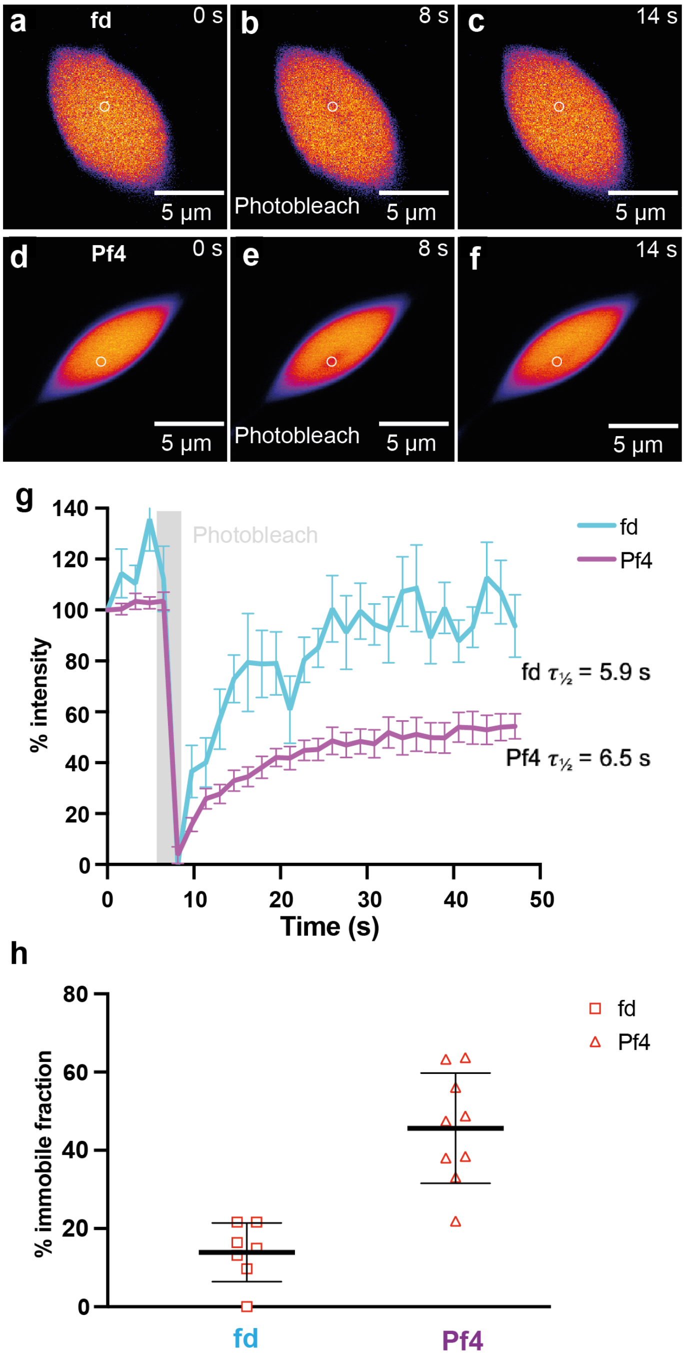

Supplementary Figure 8: Fluorescence recovery after photobleaching (FRAP) for Pf4 and fd tactoids.

a-f) Fluorescence images of tactoids before (left), immediately after (middle), and 6s after photobleaching for fd **(a-c)** and Pf4 **(d-f)** tactoids. **g)** Fluorescence recovery curves for fd (n=7) and Pf4 (n=8), normalised to fluorescence at 0 s. The resulting half-life of recovery is shown. **h)** Immobile fraction of fd and Pf4 tactoids calculated from FRAP experiments, with mean and standard deviation indicated. Source data for graphs are provided as a Source Data file.

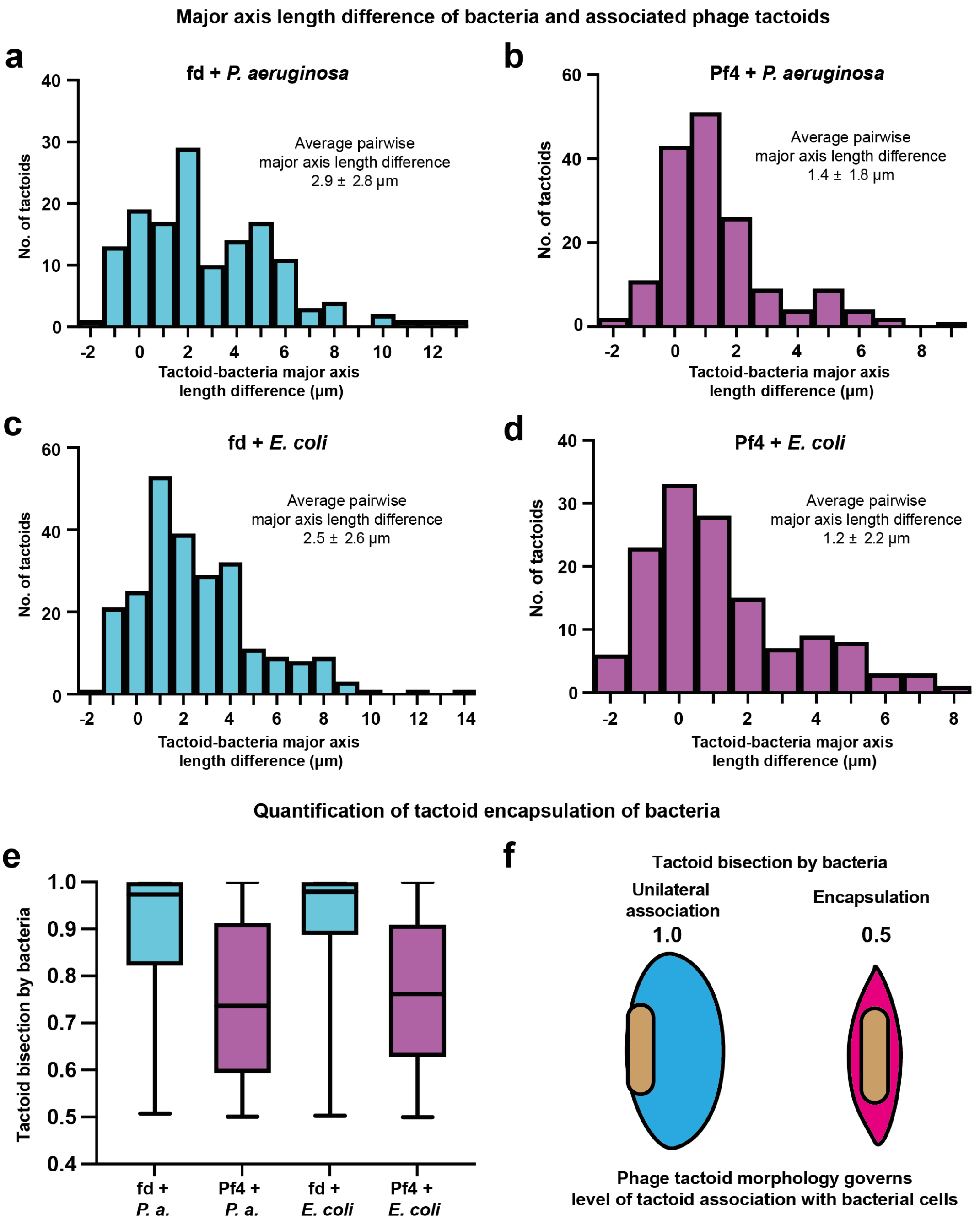

Supplementary Figure 9: Quantification of phage tactoid association with bacterial cells.

a-d) Histogram of the major axis length difference between bacterial cells and associated tactoid from semi-automated segmentation of images (Figure 5). Mean and standard deviation are reported from three independent experiments, **a)** fd + *P. aeruginosa* (n=144), **b)** Pf4 + *P. aeruginosa* (n=163), **c)** fd + *E. coli* (n=244) and **d)** Pf4 + *E. coli* (n=137). Fd + *P. aeruginosa* versus Pf4 + *P. aeruginosa*, **** $P_{value} < 0.0001$. fd + *E. coli* versus Pf4 + *E. coli*, **** P < 0.0001. Pf4 + *P. aeruginosa* versus Pf4 + *E. coli*, no significant difference, $P_{value}= 0.4999$. Fd + *P. aeruginosa* versus fd + *E. coli*, no significant difference, $P_{value}= 0.2590$. Pf4 + *P. aeruginosa* versus fd + *E. coli*, **** $P_{value} < 0.0001$. Fd + *P. aeruginosa* versus Pf4 + *E. coli*, **** $P_{value} < 0.0001$. **e)** Plot showing type of association of phage tactoids with bacterial cells from semi-automatic segmentation of images. This measurement was obtained by measuring the

bifurcation of the major axis of the segmented bacterial shape of the tactoid. The ratio of the resulting two shapes was then calculated. **f)** Values of 0.5 indicate encapsulation of bacteria by tactoids whereas values of 1 indicate unilateral association of tactoids with bacteria. All p-values were calculated using an unpaired t-test. Source data for graphs are provided as a Source Data file.

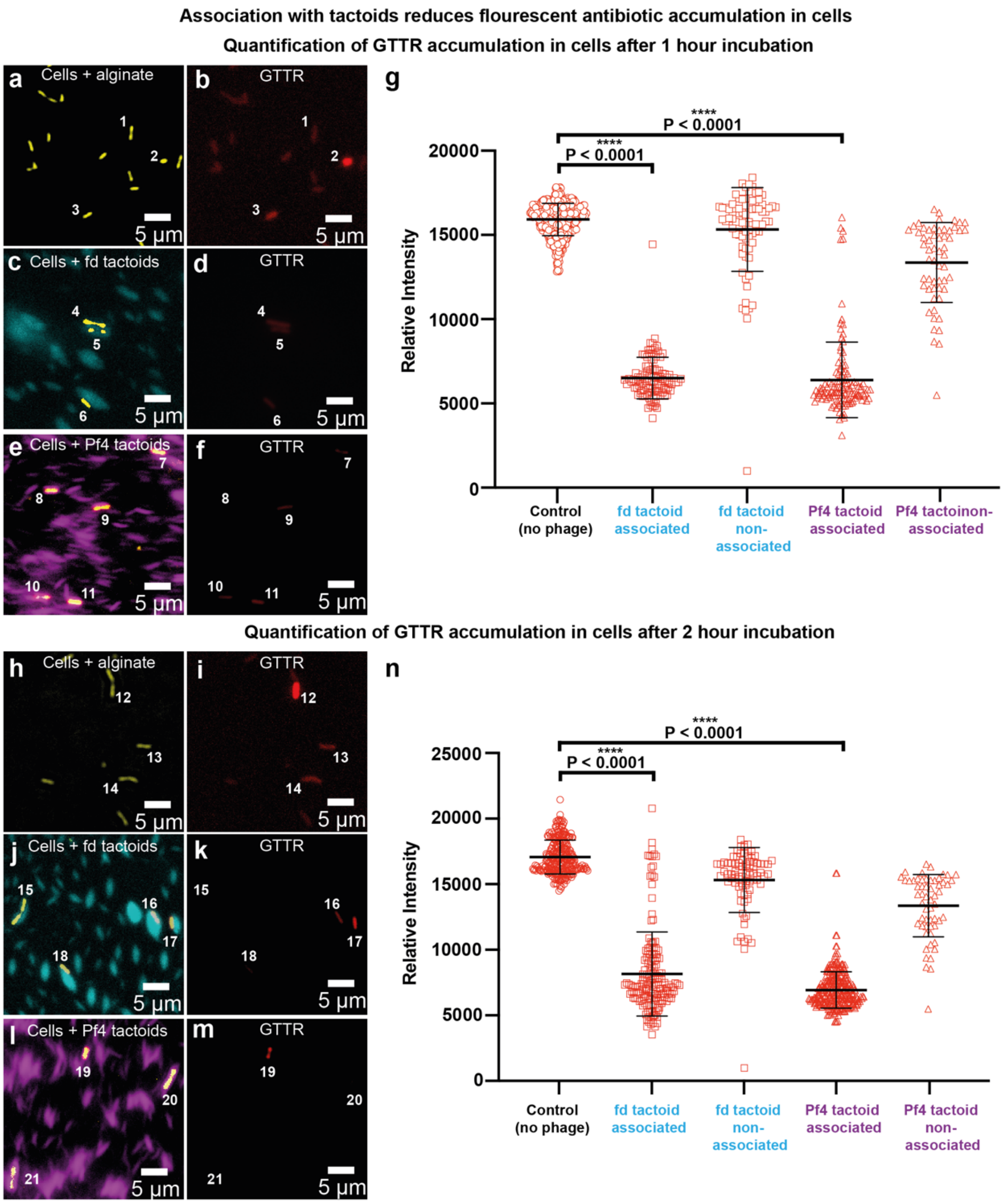

Supplementary Figure 10: Association with tactoids protects bacterial cells from antibiotic uptake.

Fluorescence and light microscopy images of Alexa 488-labelled Pf4/fd phage tactoids incubated with Texas Red-labelled gentamicin (GTTR) for **(a-f)** 1 hour and **(h-m)** 2 hours. Shown are Alexa488 phage signal (Cyan – fd, Magenta – Pf4) and pseudocoloured cells (yellow) as determined through brightfield light microscopy (left), and Texas Red signal corresponding to uptake of the fluorescently labelled antibiotic GTTR by cells (right). Numbering indicates site of the same cells in corresponding images. Images are representative of 30 images taken over three biological replicates (Control-1 hour, n=508 cells, fd associated-1hour, n=111, fd non-associated-1 hour, n=82, Pf4 associated-1 hour, n=120, Pf4 non-associated-1 hour, n=57, Control-2 hour, n=276 cells, fd associated-2 hour, n=165, fd non-associated-2 hour, n=82, Pf4 associated-2 hour, n=294, Pf4 non-associated-1hr, n=67 **g)** and **n)** Plots quantifying GTTR uptake after 1 hour and 2 hours respectively in conditions indicated on the x-axis. Bar shows the mean of three independent experiments and error bars represent standard deviation. Association with both Pf4 and fd tactoids results in significantly

reduced antibiotic uptake as compared to a control with no phage (fd $P_{value} < 0.0001$, Pf4 $P_{value} < 0.0001$), and as compared to cells in the same sample that are not tactoid-associated (fd $P_{value} < 0.0001$, Pf4 $P_{value} < 0.0001$). All p-values were calculated using an unpaired t-test. Source data for graphs are provided as a Source Data file.

Supplementary Tables 1-2

Supplementary Table 1: Data collection and processing statistics for fd capsid structure.

**Data collection and processing**	[EMDB 16657, PDB 8CH5]
Microscope	Krios Titan G3
Magnification	81,000
Voltage (kV)	300
Electron exposure (e^{-}/Å^2)	53.9
Defocus range (μm)	-1 to -3
Pixel size (Å)	1.092
Symmetry imposed	C5 and Helical, final: 35.46°,16.62 Å
Initial particle images (no.)	1,514,504
Final particle images (no.)	84,458
Map resolution (Å)	3.2
FSC threshold	0.143
Map resolution range (Å)	3.2-4.0
**Refinement**	
Initial model used (PDB code)	n/a
Model resolution (Å)	3.1/3.7
FSC threshold	0.143/0.5
Model resolution range (Å)	n/a
Map sharpening *B* factor (Å^2)	-56
Model composition	
Non-hydrogen atoms	16,800
Protein residues	2,250
Ligands	0
B factors (Å^2)	
Protein	79.94
Ligand	n/a
R.m.s. deviations	
Bond lengths (Å)	0.003
Bond angles (°)	0.443
Validation	
MolProbity score	1.47
Clashscore	8.82
Poor rotamers (%)	0
Ramachandran plot	
Favored (%)	100
Allowed (%)	0
Disallowed (%)	0

Supplementary Table 2: Molecular dynamics simulation parameters

Simulated System	Source Structure	Approach	Forcefield	Box Volume (nm^3)	Total atom number	Water molecule number	Ion number Na/Cl	Salt concentration [NaCl] (mM)	Number of replicates	Simulation time (ns)*
Pf4 capsid	PDB: 67UQ	Atomistic MD	CHARMM-36	12.7	208530	63125	227/193	150	4	250
Fd capsid	This study	Atomistic MD	CHARMM-36	12.6	203361	61174	187/192	150	4	250

*Simulation time is per replicate
