# Supplementary material for: Biophysical basis of filamentous phage tactoid-mediated antibiotic tolerance in P. aeruginosa
Source: Nat Commun. 2023 Dec 19;14:8429. doi: 10.1038/s41467-023-44160-8 (PMC10730611; doi:10.1038/s41467-023-44160-8)
Supplement: Supplementary file 3 — Description of additional supplementary files [file 41467_2023_44160_MOESM3_ESM.pdf]

<u>Description of additional supplementary files</u>

Supplementary Movie 1: Cryo-EM map (10 s away from the mean) and atomic model (ribbon depiction) of the bacteriophage fd.

Supplementary Movie 2: Electron cryotomograms of tactoids formed by Pf4 (upper) and fd (lower).

Supplementary Movie 3: FRAP experiments on fd and Pf4 tactoid. An fd and Pf4 tactoid was photobleached multiple times and fluorescence recovery observed. Photobleaching events are labelled. Video is 46 seconds in length (yellow – high signal, blue – low signal).
