# Supplementary material for: Biophysical basis of filamentous phage tactoid-mediated antibiotic tolerance in P. aeruginosa
Source: Nat Commun. 2023 Dec 19;14:8429. doi: 10.1038/s41467-023-44160-8 (PMC10730611; doi:10.1038/s41467-023-44160-8)
Supplement: Supplementary file 7 — Reporting Summary [file 41467_2023_44160_MOESM7_ESM.pdf]

nature portfolio

Corresponding author(s): Tanmay Bharat, Abul Tarafder

Last updated by author(s): Nov 27, 2023

Reporting Summary

Nature Portfolio wishes to improve the reproducibility of the work that we publish. This form provides structure for consistency and transparency in reporting. For further information on Nature Portfolio policies, see our Editorial Policies and the Editorial Policy Checklist.

Statistics

For all statistical analyses, confirm that the following items are present in the figure legend, table legend, main text, or Methods section.

n/a	Confirmed	
☐	☒	The exact sample size (*n*) for each experimental group/condition, given as a discrete number and unit of measurement
☐	☒	A statement on whether measurements were taken from distinct samples or whether the same sample was measured repeatedly
☐	☒	The statistical test(s) used AND whether they are one- or two-sided<br>*Only common tests should be described solely by name; describe more complex techniques in the Methods section.*
☒	☐	A description of all covariates tested
☒	☐	A description of any assumptions or corrections, such as tests of normality and adjustment for multiple comparisons
☐	☒	A full description of the statistical parameters including central tendency (e.g. means) or other basic estimates (e.g. regression coefficient) AND variation (e.g. standard deviation) or associated estimates of uncertainty (e.g. confidence intervals)
☐	☒	For null hypothesis testing, the test statistic (e.g. *F*, *t*, *r*) with confidence intervals, effect sizes, degrees of freedom and *P* value noted<br>*Give P values as exact values whenever suitable.*
☒	☐	For Bayesian analysis, information on the choice of priors and Markov chain Monte Carlo settings
☒	☐	For hierarchical and complex designs, identification of the appropriate level for tests and full reporting of outcomes
☒	☐	Estimates of effect sizes (e.g. Cohen's *d*, Pearson's *r*), indicating how they were calculated

Our web collection on statistics for biologists contains articles on many of the points above.

Software and code

Policy information about availability of computer code

Data collection	EPU 2, SerialEM 3, Zeiss Zen Blue
Data analysis	RELION 3.1, COOT 0.9, PHENIX 1.2, MATLAB 2021b, MotionCor2, CtfFind4, UCSF ChimeraX 1.3, Fiji 1.0, GraphPad Prism 9, VMD 1.94, PyMol 2.52, Gromacs 2021.3, PyLipID, MDAnalysis, Plotly 5.11.0

For manuscripts utilizing custom algorithms or software that are central to the research but not yet described in published literature, software must be made available to editors and reviewers. We strongly encourage code deposition in a community repository (e.g. GitHub). See the Nature Portfolio guidelines for submitting code & software for further information.

Data

Policy information about availability of data

All manuscripts must include a data availability statement. This statement should provide the following information, where applicable:

- Accession codes, unique identifiers, or web links for publicly available datasets
- A description of any restrictions on data availability
- For clinical datasets or third party data, please ensure that the statement adheres to our policy

The cryo-EM density map of the fd phage capsid generated in this study has been deposited in the Electron Microscopy Databank (EMDB) under the accession number EMD-16657 [https://www.ebi.ac.uk/emdb/EMD-16657]. The corresponding atomic coordinates are deposited in the Protein Data Bank (PDB) under accession code 8CH5 [https://doi.org/10.2210/pdb8ch5/pdb]. The atomic coordinates of other phage capsids used for comparison are available on the PDB under accession codes 6TUP [https://doi.org/10.2210/pdb6tup/pdb] (cryo-EM structure of Pf4), 6A7F [https://doi.org/10.2210/pdb6a7f/pdb] (cryo-EM structure of IKe

phage), 2HI5 [https://doi.org/10.2210/pdb2hi5/pdb] (previous fd phage cryo-EM model), 2C0X [https://doi.org/10.2210/pdb2c0x/pdb] (fibre diffraction/ssNMR model of fd), 1IFI [https://doi.org/10.2210/pdb1ifi/pdb] (fibre diffraction model of fd), 1NH4 [https://doi.org/10.2210/pdb1nh4/pdb] (ssNMR model of fd). MD data is available on Zenodo (doi.org/10.5281/zenodo.10175088). Source data are provided with this publication. All other data are available from the corresponding authors upon request.

Research involving human participants, their data, or biological material

Policy information about studies with human participants or human data. See also policy information about sex, gender (identity/presentation), and sexual orientation and race, ethnicity and racism.

Reporting on sex and gender	No human participants were used in this study.
Reporting on race, ethnicity, or other socially relevant groupings	No human participants were used in this study.
Population characteristics	No human participants were used in this study.
Recruitment	No human participants were used in this study.
Ethics oversight	No human participants were used in this study.

Note that full information on the approval of the study protocol must also be provided in the manuscript.

Field-specific reporting

Please select the one below that is the best fit for your research. If you are not sure, read the appropriate sections before making your selection.

[x] Life sciences [ ] Behavioural & social sciences [ ] Ecological, evolutionary & environmental sciences

For a reference copy of the document with all sections, see nature.com/documents/nr-reporting-summary-flat.pdf

Life sciences study design

All studies must disclose on these points even when the disclosure is negative.

Sample size	Samples sizes were not pretermined. For light microscopy experiments, the number of images acquired yielded statistically significant results, balanced with practical limitations in acquisition times. Light microscopy assays were conducted in triplicate. Antibiotic protection assays were conducted in triplicate in agreement with previous literature, yielding statistically significant results. For electron microscopy experiments, sufficient micrographs were acquired to enable structural solution. Molecular dynamics experiments were
Data exclusions	No data were excluded, apart from exclusion of low-quality particles via classification during cryo-EM helical reconstruction, in agreement with typical cryo-EM processing workflows.
Replication	Apart from cryo-EM image processing, each experiment was performed at least in triplicate, with each measurement being independent. Conventional replication is not normally performed during cryo-EM single particle analysis, which is an averaging method. The samples was screened multiple times, showing equivalent particle morphology in all cases. During processing, the dataset is split into independent half-sets during refinement to avoid overfitting. For all experiments other than cryo-EM image processing, all attempts at replication were successful.
Randomization	Assignment of cryo-EM particles into random half-sets during image processing was performed as per field standards. Cryo-EM images were acquired automatically. Light microscopy images were acquired at random positions and evaluated using semi-automated segmentation procedures. Samples were otherwise allocated into random groups.
Blinding	No blinding was performed as per typical workflows in the experiments. In cryo-EM, image acquisition and processing are automated. In light microscopy, images were acquired randomly and image processing is semi-automated. In other experiments, blinding is not commonly performed as per previous literature.

Reporting for specific materials, systems and methods

We require information from authors about some types of materials, experimental systems and methods used in many studies. Here, indicate whether each material, system or method listed is relevant to your study. If you are not sure if a list item applies to your research, read the appropriate section before selecting a response.

Materials & experimental systems

n/a	Involved in the study
☒	☐ Antibodies
☒	☐ Eukaryotic cell lines
☒	☐ Palaeontology and archaeology
☒	☐ Animals and other organisms
☒	☐ Clinical data
☒	☐ Dual use research of concern
☒	☐ Plants

Methods

n/a	Involved in the study
☒	☐ ChIP-seq
☒	☐ Flow cytometry
☒	☐ MRI-based neuroimaging

Plants

Seed stocks

No plants were used in this study.

Novel plant genotypes

No plants were used in this study.

Authentication

No plants were used in this study.
